# Feasibility of a safe innovation framework for crop breeding

**DOI:** 10.1080/21645698.2025.2524236

**Published:** 2025-06-26

**Authors:** Marc Groenen, Gijs W. Spaans, Lianne. M.S. Bouwman, Gijs A. Kleter, Jan Pieter van der Berg

**Affiliations:** Wageningen Food Safety Research (WFSR), Wageningen University & Research, Wageningen, The Netherlands

**Keywords:** Biotechnology, plant breeding, safe innovation, safe-by-design, safety framework

## Abstract

Biotechnological innovations accelerate the development of new plant varieties at an ever-increasing pace. A framework for safe innovation can create opportunities for the integration of safety considerations into each stage of the lifecycle of these products. Through stakeholder engagement, we discussed their views, preferences, as well as perceived challenges surrounding safe innovation and innovative biotechnological tools in the plant breeding sector. Furthermore, five scenarios for safe innovation frameworks were presented to them. Stakeholders from the Dutch plant breeding sector favored a self-regulated safety scenario, allowing the plant breeding sector to develop best practices with guidance from the government and independent researchers. Based on the stakeholders’ views, we developed a draft voluntary framework for safe innovation named “Safe Innovation in Plant breeding (SIP) Framework,” which is grounded in safe innovation principles, and focuses on food safety in particular.

## Introduction

Over the past century, major innovations in the plant breeding sector have allowed for the development of new plant varieties with favorable traits at an ever-increasing pace. Whilst this contributes to food security, it should ideally also promote their sustainability and safety. To ensure the safe commercialization of new plant breeds developed by means of biotechnological techniques, regulatory frameworks are in place, often based on scientific risk assessment.^1^ However, these regulatory frameworks may not always be fit for purpose when considering emerging technologies, both in biotechnology and beyond, in terms of, for example, lack of coverage or not being risk-proportionate. As innovation often precedes specific regulation, the application of novel technologies for crop developing purposes may be present before detailed regulations or guidelines are in place covering (food) safety aspects. A case in point are techniques such as cisgenesis, genome editing, and epigenome editing, which have been applied in development of experimental genetically improved crops for over two decades yet the pertinent legislation is still subject to deliberation and amendments in many countries.

Particularly, potential food safety aspects should be evaluated when novel crop varieties are developed using emerging technologies which may not yet be covered by regulations. One approach to foster safety is through a safe innovation framework, including a structured procedure or set of guidelines designed to promote innovation within an organization while ensuring that future challenges are managed effectively.^2^ Furthermore, such a safe innovation framework can be broadly based on the principles of Safe-by-Design, or related principles, which emphasize integrating safety considerations across the entire lifecycle of a process or product. It aims to anticipate and mitigate potential risks and hazards upfront, starting at the design stage rather than addressing them as an afterthought or during operation.

Safety frameworks benefiting crop breeding innovation that are kept up to date with technological developments may help regulators and plant breeders alike.^3^ It is anticipated that the development of additional internal safety protocols in the plant breeding sector, such as a framework that incorporates Safe-by-Design principles, may complement and/or strengthen existing plant breeding practices. Thus, safety protocols can help to maintain an excellent standard of food safety when new techniques are applied in plant breeding. In addition, data collected voluntarily at an early stage of product development according to a safety protocol can be used later in the process for preparing a safety dossier, when necessary. Such a dossier may be beneficial when potential regulatory adjustments are made for any methods or outcomes not yet covered by current regulation.

Thus, the purpose of this research is to investigate, through stakeholder engagement, if and in what form the Dutch plant breeding sector can implement a safety framework which can benefit crop breeding innovation in the Netherlands. This sector is renowned for its innovation and expertise in developing new crop varieties. A case can therefore be made for this sector to discuss safety strategies for novel breeding techniques that are not yet covered by legislation. Within the context of this paper, “emerging technologies” refers to future breeding techniques, genomic or otherwise, that would fall outside the scope of current regulation. Here, we focus in particular on crop breeding innovation utilizing such emerging technologies and the potential benefits a safety framework may yield regarding product safety and potentially adapting regulatory compliance. A concept framework for safe innovation was developed with input gathered during interviews and workshops with relevant stakeholders from the Dutch plant and seed breeding sector, governmental organizations, and interest groups. It is important to note that whilst we focus on food safety aspects, the proposed framework can also benefit other areas of safety, such as environmental safety.

## Theoretical Background

### Plant Breeding Innovation

Innovation is moving rapidly in the plant breeding sector and biotechnological innovations applied to plant breeding encompass a diverse array of techniques. Genome editing using CRISPR-Cas, for instance, enables precise and targeted modification of plant genomes without the introduction of foreign DNA.^4^ This technology offers immense potential in accelerating the development of new crop varieties.^5^ With the wide range of tools available, plant breeders have many opportunities to create new crop varieties in ways that are not possible in the same time frame using more conventional plant breeding methods. Examples of recent technological advances include *de novo* domestication of wild plants based on gene editing,^6^ as well as rootstock grafting using a non-genetically modified (GM) scion on a GM rootstock. Rootstock grafting results in epigenetic changes that give desired traits in the scion as well as in the harvested fruits.^7,8^

In part, integration of these latest technological developments has revolutionized the breeding process. With genome editing, plant breeders and scientists are able to potentially improve a large variety of phenotypical characteristics of crops. The integration of biotechnological tools into plant breeding programs has opened new avenues toward achieving sustainable and resilient agriculture capable of addressing the global challenges of food security, climate change, and population growth.^9^ As with conventional plant breeding practices, innovative new plant breeding techniques such as CRISPR-Cas might yield unintended effects. For example, such unintended effects can be genetic insertions or deletions in regions of the genome other than the on-target sequence.^10^ However, these new techniques still yield unintended effects to lesser extent than technologies such as random mutagenesis as well as natural variation. In fact, improvement of the CRISPR-Cas delivery method, guide RNA, and the Cas protein can increase gene editing efficiency and reduce occurrence of off-target effects.^11^ Future biotechnological innovations can, nevertheless, introduce complexities that may require specific safety considerations to ensure that unintended effects are minimized.

### Regulation of Modern Biotechnology

#### Regulation in the European Union

In the EU, genetically modified organisms (GMOs) used in plant breeding are regulated according to Directive 2001/18/EC on the deliberate release into the environment of genetically modified organisms. Depending on the use of the developed plant, Regulations (EC) No 1829/2003 on GM food/feed and (EC) No 1830/2003 on labeling and traceability may also apply. “New genomic techniques” (NGTs) are broadly defined by some EU institutions as any technique capable of changing genetic material which has evolved after the publication of the EU Directive 2001/18/EC (defining GMOs). Yet the discussions surrounding NGTs focus frequently on those introducing small edits to the DNA, RNA, or epigenome, and which do not introduce foreign DNA.^12^ Currently in the EU, all plants developed using these new techniques are to be considered and labeled as GMO. Targeted mutations created by, *e.g*., gene editing, however, are difficult, if not impossible, to distinguish from natural genetic variation and mutations caused by conventional mutagenesis techniques that are exempt from GMO regulation.^13^ Moreover, the European Commission carried out a study in 2021, which concluded that the existing GMO regulation is outdated and not fit for purpose, in particular with regards to the status of plants developed using NGTs under EU law. Therefore, the European Commission called for more future-proof and resilient legislation.^14^ The European Commission followed this up with a proposal for an amendment to the GMO regulation in 2023, which it presented to the European Parliament. This proposal defined a “NGT plant” as “*a genetically modified plant obtained by targeted mutagenesis or cisgenesis, or a combination thereof, on the condition that it does not contain any genetic material originating from outside the breeders’ gene pool that temporarily may have been inserted during the development of the NGT plant*.”^15^ The Parliament’s amendments of the proposal aimed to address concerns of Members of Parliament over, for instance, the protection of plant breeders’ rights *versus* the patentability of NGT crops, and the traceability thereof in order to provide consumers the freedom of choice. In addition, although herbicide-tolerant crops might help address certain environmental aims, the changed use of herbicides could also be potentially harmful, for which reason they should never be fully exempt from evaluation requirements. More technical details that were amended included a more extensive description of the criteria for equivalence of an NGT crop to conventional plants, particularly the molecular characteristics, such as insertions, deletions, or inversions of a given number and/or size.^16^ As of this writing, the proposal has been supported by the Council of the EU, which also made several suggestions regarding environmental and health objectives and raised some concerns regarding patenting during its mandate to negotiate between Commission, Parliament and Council.^17^ This mandate allows the European Parliament to negotiate between Commission, Parliament and Council before they adopt any final text of the proposed legislation.

#### Summary of the Regulatory Landscape Outside the EU

Since the conception of biotechnology in agriculture, governments have drafted regulations to ensure that biotechnology derived products placed on the market are safe.

##### USA

Already in 1986, the Coordinated Framework for Regulation of Biotechnology was established in the USA. The framework clarified the roles and responsibilities of governmental agencies (EPA, FDA, and USDA) regarding the regulation of products of biotechnology, and this division of tasks still exists. The US Department of Agriculture’s Animal and Plant Health Inspection Service (USDA APHIS) primarily investigates whether released or transported “genetically engineered” organisms may become plant pests or diseases. “Genetic engineering” is any modification of the genome that would not fall within the scope of conventional breeding. Whether the engineered organism is of concern depends on the nature of the host and the introduced genetic materials and traits. Developers can enquire with USDA APHIS whether the products they are developing are indeed regulated, based on relevant information provided. The outcomes of such consultations are published on the USDA’s website.^18^

The US Environmental Protection Agency (US EPA) will consider traits that enable the plant to resist pests and diseases, that is, so-called “plant-incorporated protectants” (PIPs). US EPA has defined criteria for the exemption of certain genes coding for PIPs from regulation (under the Federal Insecticide, Fungicide, and Rodenticide Act). These exemptions include gene insertions or mutations that are introduced into native genes which occur already in the same host species or crossable wild relatives. These mutations are restricted to those from one other plant source at a time, though, being representative of what also could occur during conventional breeding.^19^

The safety of biotechnology-derived foods was considered by the US Food and Drug Administration (US FDA) in its “statement of policy” in 1992. This document focused on the risk characteristics of the modified product as the trigger for risk assessment rather than the genetic modification *per se*. Literature commonly refers to this approach as “product-based,” as opposed to the “process-based” approach for the regulation of any GM food in some other countries. The FDA has a voluntary consultation procedure in place under the Federal Food, Drug, and Cosmetics Act, allowing developers to contact the agency and receive feedback. In 2024, FDA published guidance for developers, explaining how the principles of the 1992 statement would apply to genome edited foods. This FDA guidance stated, for example, that it is the properties of the resulting food rather than the genetic change per se that may raise safety questions. Examples mentioned include increased levels of allergenic proteins, toxins, or antinutrients, or impacts on nutritional value. Moreover, the FDA guidance envisages the extension of applications of the edited food product to new uses as well as considering the possibility of introducing new genes or additional copies of intrinsic genes. Developers can consult and meet with FDA to discuss the regulatory status of their product and the safety data that are warranted. When the consultation is completed, FDA may send the developer a “no further questions” letter based on the information received. This letter is also published on the FDA’s website. FDA also encourages developers to follow a similar, parallel “New proteins consultation” procedure for non-pesticidal proteins.^20^

##### Canada

In Canada, the health ministry Health Canada has regulatory oversight over novel foods under the Novel Foods Regulation. Herein, a given food is designated as being “novel” based on the characteristics of the food that the consumer will ultimately consume. These characteristics include the technology, recombinant DNA molecules, or mutagenic substances that may have been used to obtain the food, to which the consumer itself will not be exposed. Moreover, a recognized practice in plant breeding is the recurrent crossing and selection. This way, breeders can eliminate any undesirable effects of breeding in the plant. These unwanted effects would also include, for example, any gene editor-encoding DNA temporarily introduced to effectuate the desired gene edit, as well as unintentional edits at off-target sites (the latter can also be identified using molecular techniques). Health Canada considers that gene editing technologies do not raise any concerns that would be different from other technologies. Gene edited crops should therefore be treated the same way as other crops under the Novel Food Regulation.^21^

Developers and plant breeders can seek advice from Health Canada to determine the novelty status of their product. For gene edited plants grown for food use that do not deliver *novel* foods, Health Canada has established a register under the so-called “public transparency initiative.” Here, plant developers can voluntarily provide information on such crops, provided they have not consulted with Health Canada about the status of their novelty. For example, this list mentions a gene edited *waxy* maize variety that is high in amylopectin, a particular type of starch. Because mutant *waxy* varieties already occur in nature, it is not considered novel.^22^ As for the varieties themselves, these may also qualify as “plants with novel traits” if the introduced trait is new to the cultivated varieties of Canada *and* there is a chance that the plant could have significant environmental impact in terms of weediness, impact on non-target organisms and biodiversity, the potential to become a plant pest, and the impact of vertical gene transfer to wild plants. As for novel foods, the focus here is on the trait and not the method used to introduce it. Plants with foreign DNA introduced will require authorization in any case. Moreover, any new plant with a commercially viable herbicide tolerance has to be notified as well. Developers can consult with the Plant Biotechnology Office of the Canadian Feed Inspection Agency, which oversees this regulatory process, to check the novelty status of their plant.^23^

##### Australia

The environmental release of GM plants and other GMOs falls under the Australian Gene Technology Act, for which the Gene Technology Regulator has regulatory oversight. This regulator has clarified which forms of gene editing applied to plants are considered genetic modification and which are not. For example, it does *not* consider a gene edited plant obtained through the “site-directed nuclease-1” (SDN-1) mechanism as a GMO. SDN-1 entails the use of a nuclease enzyme, such as CRISPR Cas9 with guide RNA, zinc-finger nucleases, or TALENS without the use of templates to guide the cellular repair of the double-strand breaks which the nuclease causes in the plant cell’s DNA. This repair commonly leads to short insertions or deletions at the cutting site. The so-called SDN-2 entails the use of templates added to the nucleases, whilst “oligonucleotide-directed mutagenesis” (ODM) involves the use of such templates alone without them. Both SDN-2 and ODM are to bring about predefined mutations in the targeted host DNA. Plants created with SDN-2 or ODM are GMOs in every case, regardless of whether the mutation caused is comparable to those in conventionally bred plants. Externally added RNA molecules are not considered gene technology. These exempted RNA molecules include double-stranded hairpin RNA molecules or antisense oligonucleotides, which are meant to silence genes by interfering with their expression. Yet the introduction of gene editing enzymes that alter the host’s mRNA molecules, for instance, is considered genetic modification. “Null-segregants” and other plants were previously temporarily genetically modified but do not contain this foreign DNA anymore. The purpose of the temporary modification may be, for example, to introduce DNA coding for the production of molecular machinery that can make certain gene edits. Such plants from which the transiently introduced transgenic DNA has been lost are not considered GMOs.^24^

Food Standards Australia New Zealand (FSANZ) oversees the regulation of GM foods in both Australia and New Zealand. It has proposed amendments to legislation on GM foods to accommodate the advent of gene editing and other new breeding technologies. The proposed amendment basically replaces the use of gene technology with the presence of “novel DNA” in the food product as the trigger for regulation, hence a shift from “process-based” to more “product-based.” “novel DNA” is very similar to “foreign DNA.” Foods from a genome edited organism but with no novel DNA present are *not* considered GM foods. The same pertains to foods from null-segregants.^25^

##### Argentina

Argentinean legislation defines GMOs as having “novel combinations of genetic material” which have been brought about by modern biotechnology. This refers to the stable introduction of any defined construct of nucleic acids into the host’s genetic material. This definition is intended to be identical to the definition of living modified organisms in the Cartagena Protocol, which is part of the international Convention on Biological Diversity. With reference this definition, developers have the possibility to consult with the government on the GMO status of their product (plant, microorganisms, animal). For this, they can submit details to an internal commission for national biosecurity (CONABIA) of the Argentinean government. This commission evaluates on a case-by-case basis whether there is indeed a novel combination of genetic material involved. In that case, the product is considered a GMO and has to follow strict regulatory procedures. If not, the product is not considered a GMO. Notably, some instances of gene editing would then be exempted from GMO regulation, whereas cisgenesis would not be exempted. Many countries in South America and beyond (*e.g.*, Japan) have followed the example posed by Argentina.^26^

#### International Harmonization of Food Safety Assessment of GMOs

The above summary shows that there are divergences amongst countries in their legislation of genetically modified products. Yet the safety assessments of foods derived from GM plants under these laws are very similar. This is the result of the international harmonization efforts in the 1980s and 1990s by international organizations such as the World Health Organization (WHO), Food and Agriculture Organization (FAO), and the Organization for Economic Cooperation and Development (OECD). This culminated into the FAO/WHO Codex Alimentarius guidelines of 2003 and extended in 2008 for the safety assessment of foods from plants derived through recombinant DNA technology.^27^

**Figure 1.**
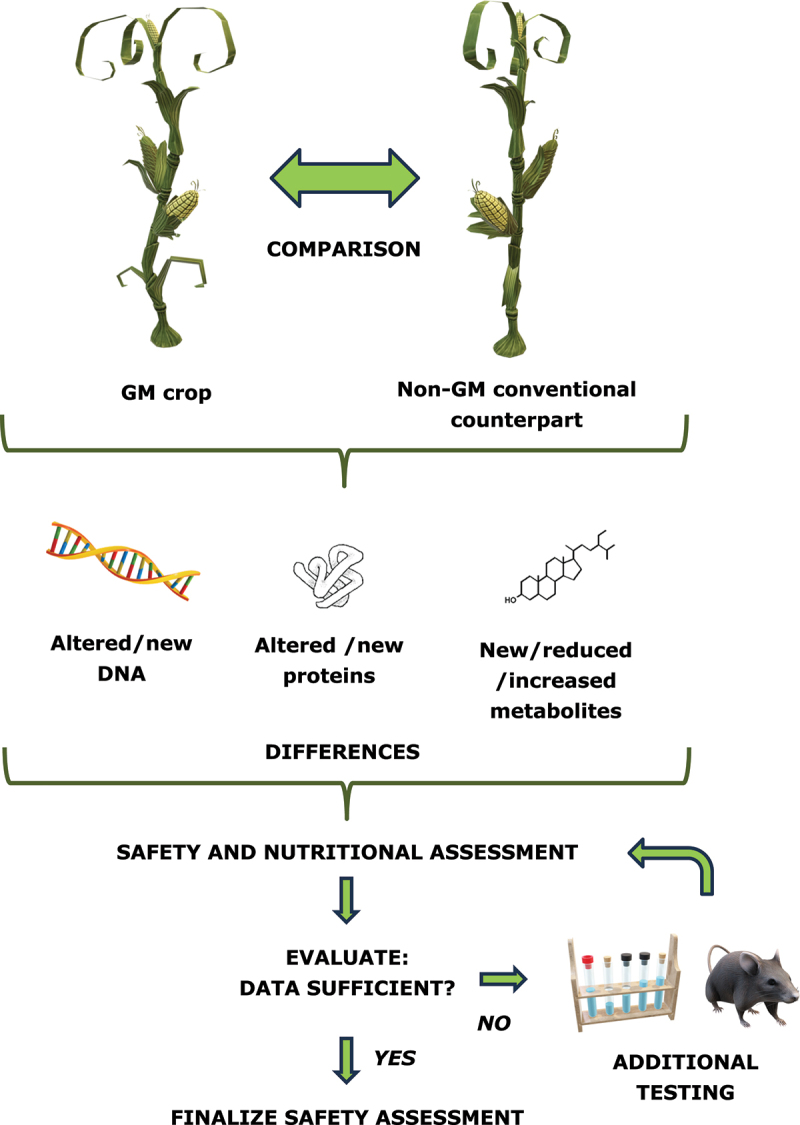
Comparative safety assessment approach.

The basic approach is that of the comparative analysis. In essence, the GM plant is compared to a conventional non-GM counterpart. This comparison entails an extensive analysis of molecular, compositional, agronomical, and phenotypical characteristics. The compositional analysis includes the measurement of the macronutrients, micronutrients (*e.g.*, vitamins, minerals), anti-nutrient, toxins, and other metabolites of interest. Based on the differences thus found, the risk assessors can decide whether further tests are needed to reach a conclusion on the safety of the GM crop (Figure 1). These tests focus on the potential toxicity, allergenicity, nutritional impact and potential for horizontal gene transfer, for example.^27^

### Frameworks for Safe Development of New Products

Safety frameworks, such as frameworks based on Safe-by-Design and its derivatives, are already explicitly being applied in other sectors, such as construction, electric engineering, food and chemical manufacture, water treatment, chemical industry or nanotechnology.^3,28^ Working according to Safe-by-Design principles means that safety is fully integrated and becomes part of the design goal during all stages of development and commercialization.^2^ Such Safe-by-Design approaches consider safety through identification of health and safety hazards at all stages of development and production, and aid in the development of a product or process which from the start has an intrinsically low risk potential. If, however, any potential hazards arise, then preventive measures may be undertaken such as minimization or elimination of the specific hazard (*e.g*., by restricting access to a certain hazard or replacing the hazard with a safer alternative). The Dutch government actively pursues Safe-by-Design within its environmental policy framework. It frames the Safe-by-Design approach to prevent harmful effects to arise from processes and products, not only covering the design stage but the whole life cycle of a given product, including its “after life.” This way, the concept also contributes to “circular by design” and “sustainable by design.” In addition, within the Organization for Economic Cooperation and Development (OECD), two Working Parties focus on the biosafety of organisms obtained through modern biotechnology and their derived foods and feeds. Both Working Parties are currently exploring the possibility to apply the “Safer Innovation Approach” to modern biotechnology. This aligns with a parallel activity within OECD in the field of safer innovation applied to nanomaterials.^29^

Many European initiatives increasingly incorporate ethical and social elements in the fields of science and technology, among which Ethical, Legal and Social Aspects and Responsible Research and Innovation (RRI) have been used most extensively. The ELSA (Ethical, Legal and Social Aspects) framework includes the view that science and technology need to better take into account ethical and societal aspects and has been used extensively since the 1990s and early 2000s, where it was incorporated in research funded by the European Commission (EC). Amongst others, this framework has been applied in the European Defence Program and the bio- and neurotechnology, nanotechnology, and robotics fields.^30^ RRI, a program to incorporate societal impact of science and technology in research projects funded by the European Union (EU), has been developed in 2011.^31^ The aim of this program was to encourage the incorporation of inclusiveness and sustainability during the development of technological innovations to enable societies to have a greater say in the policy regarding research and innovations.^32^ Various recent biotechnological trends have incited stakeholder engagement activities. A case in point is the various engagement initiatives surrounding the use of gene drives in mosquitoes for the control of malaria and other vector-borne diseases. These do not only apply to research and development but also the risk assessment of the product to be developed.^33,34^ In a more general sense, various frameworks similar to this have also been proposed for synthetic biology. As this is a vast field, perceived risks should be tackled for the specific applications.^35,36^

Certain elements of ELSA and RRI are directly linked to a Safe-by-Design approach. Amongst others, these elements consist of inclusiveness and communication with relevant stakeholders, researchers, and policy makers about the impacts of a technology being developed. Moreover, considering ethical and societal aspects will likely help increase the acceptance of new technologies by society, and should therefore be incorporated in a Safe-by-Design approach as well.^3^

## Methods

### Stakeholder Engagement

Relevant stakeholders from the plant breeding sector in the Netherlands as well as stakeholders from academia, governmental organizations, and interest groups were approached to participate in workshops and interviews on safe innovation in plant breeding with a focus on biotechnological innovations. The aim of these activities was to share information, and discuss currently employed safety strategies, potential improvements to the current situation regarding safety, as well as a general Safe-by-Design strategy. Furthermore, during these workshops, an inventory was made of the needs and potential hurdles as envisaged by participants from the plant breeding sector. Workshops were held online, by means of Microsoft Teams, and consisted of an introductory presentation by the organizers, plenary discussions, as well as discussions in break-out groups. Interactive tools such as Mentimeter (www.mentimeter.com.) and Mural (www.mural.co) were used to gather input (see Supplementary Information). A total of 27 participants took part in the two organized online workshops. These included stakeholders familiar with biotechnology from Dutch universities (2/27), governmental organizations (6/27), interest groups (3/27), as well as plant and seed breeding companies (16/27).

Following these online workshops, relevant stakeholders were approached to further discuss safe innovation in plant breeding during an interview. These interviews were used to get a better understanding of current practices during plant breeding and the technologies that are currently employed by stakeholders and to explore whether there is an interest in utilizing biotechnological innovations. Moreover, potential safety strategies as well as actual safety measures already employed to aid in guaranteeing the safety of novel plant varieties and plant products were discussed (see Supplementary Information for interview material). Five scenarios of potential safety frameworks were introduced and discussed with the participants (Table 1). Additionally, more general questions were addressed, including developments in plant biotechnology, potential safety issues, and current internal protocols used in plant breeding. A total of 11 stakeholders contributed to interviews on this topic, eight experts from plant breeding companies, two representatives from governmental organizations, and one expert from an interest group. Results from the interviews formed the basis for the development of the proposed framework for safe innovation presented in this paper.

**Table 1.** Safety scenarios for implementation of safe innovation in the plant breeding sector, discussed with stakeholders during interviews.

Scenario	Applicability “scope”	Potential additional burden	In line with or compatible with current strategy?
Safe-by-Design	From concept to commercialization	Possible, if the security strategy deviates significantly from current internal protocols	Adaptable, depending on compliance with currently used safety protocols
Self-regulated safety	During research and development process	Possible, depending on agreement with current internal protocols (*e.g*., Codex Alimentarius, HACCP)	Adaptable, and perhaps in line with current strategy
Safety culture	Safety awareness in the organization, including research/development (for example according to ISO standards), broader than just safety during the development of new products	To some extent, whilst partial fulfilment may already have been achieved through company-wide “safety thinking” according to ISO standards	In accordance with current standards, such as ISO
Design thinking	From concept to commercialization, incl. prototyping according to design thinking stages	Possibly, may require different internal attitude and mindset	Adaptable, depending on agreement with the current situation and possibly a different mindset
Current situation	Security strategy according to internal protocols, which comply with applicable food safety standards, seed laws, etc.	No, continuation of current situation, codified in Codex Alimentarius, ISO, and HACCP guidelines	Not applicable

## Results

### Safe Innovation Scenarios

Stakeholders from the Dutch plant breeding sector shared their opinions on the five possible scenarios for implementation of safe innovation in plant breeding (Table 1). Stakeholders had a preference for scenarios that provide clarity, in particular those that place the responsibility with the sector such as with the self-regulated safety example. Below, stakeholders’ opinions as well as an aggregated ranking (Table 2) of the scenarios are described.

**Table 2.** Aggregated ranking of safety scenarios for safe innovation by stakeholders during interviews.

Ranking*, number of times ranked No. 1, and average score	Scenario	Reasoning
Most preferred 5/11, avg. score: 1.3	Self-regulated safety	It is most beneficial for companies to be able to adopt their own regulations. These are nevertheless based on clear guidance from the government comprising of a combination of different voluntary and supportive measures.
5/11, avg. score: 1.7	Current situation	For companies the safety of their products is the most important. This is out of the companies’ own interest, as mistakes cannot be afforded. The current situation provides clarity and ensures the safety of the products.
2/11, avg. score: 2.3	Safety culture	This scenario represents safety awareness and commitment throughout the organization. When developing new plant varieties it is important that staff know what they are doing and that they know the processes and safety requirements.
2/11, avg. score: 3.2	Safe-by-Design	A Safe-by-Design approach is not supported as it is seen that it brings regulatory obligations, which would equate to the introduction of additional regulation.
Least preferred 0/11, avg. score: 3.8	Design thinking	Design thinking seems to focus too much on a problem to be solved.

*Stakeholders could assign the same ranking to multiple scenarios. Three stakeholders gave a shared No. 1 ranking to two scenarios, resulting in 14 No. 1 rankings overall.

#### Safe-By-Design

The proposed Safe-by-Design scenario consists of a framework that combines safety principles and principles of trusted environments.^3^ Safety principles can be implemented in three ways:

- by providing best practices for plant breeders,
- by including information on what to test for and how to deal with potential risks and vulnerabilities, and
- by listing strategies to mitigate or prevent potential hazards. These safety principles are to be implemented at every stage of the product’s lifecycle to improve its safety.

Authorities also play an important role in implementing principles of trusted environments. These principles enable a free dialogue between plant breeders and risk professionals employed by governmental organizations. Such consultations should be regularly conducted and allow developers to further ensure the development of safe new plant varieties. They also enable regulators to get information on current developments in the technological field. Policy makers can use this information to increase awareness of potential necessary adaptations in regulation needed to keep up with technological innovations.

During the interviews, stakeholders made it clear that they preferred the government to promote Safe-by-Design by providing practical guidelines and toolboxes. However, they did not support a Safe-by-Design approach if it would be part of obligatory safety legislation. Such mandatory regulations were considered unnecessary by the stakeholders owing to the “low risk profile” of the technologies used. Nevertheless, it was mentioned that consumers’ trust in these technologies could benefit from the adoption of a safety strategy. Moreover, 6 out of 11 of the stakeholders voiced their willingness to participate in consultations with external experts. Although interviewees replied that transparency about these consultations would be important, since being open about such communications (*e.g*., involved participants, and potential interests), may increase trust and thereby benefit social acceptance. Such consultations would be particularly interesting for small and medium-sized enterprises (SMEs), as consultation provides them with clarity on the regulatory status of crop breeding innovations and what information is necessary to be included in an application.

#### Self-Regulated Safety

In the self-regulated safety scenario, independent researchers together with plant breeders whilst risk professionals create best practices for safe innovation, which incorporate safety principles. These best practices are agreed upon and self-regulated by the plant breeding sector. Moreover, the best practices are regularly updated based on technological developments. These practices would be complementary to the existing legal and regulatory provisions for the plant breeding sector.

This approach shares similarities with parts of existing processes, of which value for cultivation and use (VCU) is a good example, in particular for potato varieties. The VCU was developed by stakeholders from the plant breeding sector, which included, among others, breeders, researchers, and farmers. Part of this VCU are criteria for the safety of new plant varieties.^37^ For new potato breeds, for instance, this includes the testing of glycoalkaloid levels, production yield, as well as resistance to phytopathogens such as potato virus Y and *Phytophthora infestans* (foliage blight).^38^

During the interviews, stakeholders generally had a positive attitude toward the self-regulated safety scenario. It was noted that this scenario would be most beneficial for companies when governments provide clear guidance consisting of different voluntary and supportive measures. Companies are then able to voluntarily adopt these guidelines and accommodate them into their own regulations. Moreover, stakeholders stressed that for them, this self-regulated safety scenario closely resembles the current situation within their companies since, out of safety precaution, rules within these companies are often already more strict than existing regulations.

#### Safety Culture

The third scenario consists of a general safety culture as described by the FAO/WHO Codex Alimentarius Commission.^39^ The introduction of this concept by Codex in 2021 was based on the recognition that food safety is increased by more awareness and improving behavior of staff within food facilities. Codex Alimentarius is an organization that develops internationally harmonized standards for food products, for instance concerning principles of food hygiene. Adherence to such safety culture guidelines will have to be evaluated by accrediting bodies based on a set of criteria gauging the management and staff’s commitment. Within the EU, this has been accommodated as an amendment to the Food Hygiene Regulation (EC) No 852/2004. Major food-producing companies have already included food safety culture into their corporate governance models and several governments also have schemes in place to evaluate safety culture. Criteria include, for instance; the values and mission of a company, the attitudes of managers, the amount of education on this topic within a company, the consistency of actions and leadership, adaptability toward improving food safety, and risks awareness throughout the organization. Plant breeders can follow procedures as defined by national standards bodies, such as ISO or the NEN (the Royal Netherlands Standardization Institute [*e.g*., the safety culture ladder of NEN]). Official standards defined by these organizations include best practices and guidance to help create a safety culture, and compliance with the requirements of these standards is necessary for certification. Businesses can demonstrate that requirements are adhered to through, for instance, audits, and record keeping.

During the interviews, stakeholders generally viewed this scenario as neutral to positive. Stakeholders considered the implementation of safety culture elements as well as risk awareness throughout the organization to be important.

#### Design Thinking

In the design thinking scenario, the end user’s preferences and the context of the application are at the focal point and efforts are made toward understanding the prospective users of the product or service being developed. “Design” currently has acquired a much broader meaning than just the process of technical design of a concrete product: some argue that it is actually any attempt to find a solution to a problem using novel or innovative ideas, hence everybody could be a “designer.” In many models of design-thinking, different stages are discerned,^40^ starting with:

- “Empathize” (understand the problems and needs of the users) before proceeding to the next stage.
- “Define” (interpret the findings of the previous stage and define the problem).
- “Ideate” (generate as many solutions as possible to the problem, “think out of the box”).
- “Prototype” (create simple versions of the ideas, iteratively test a working prototype for further improvement or rejection, explore acceptance and technicalities).
- “Test” (have the selected product or service tested by true end users, of which the outcomes may necessitate revisiting previous steps).

During the interviews, design thinking was the least preferred scenario. Some stakeholders reasoned that design thinking focuses too much on a problem to be solved, what they considered to be irrelevant to plant breeding.

#### Current Situation

The last scenario concerns maintaining the status quo, meaning no new frameworks or guidelines will be developed. No specific best practices for safe innovation will be generated. Current internal protocols suffice for the development of new commercial plant varieties, in which the plant breeding sector adheres to existing legal requirements and regulations.

During the interviews, stakeholders positively viewed this current situation scenario as it provides clarity. Stakeholders argued that, from a business economic perspective, it is of utmost importance for the company to develop a safe product. They conveyed that the safety of the products is thus already guaranteed through a form of self-regulation based on the company’s own interests.

In general, the safety scenario most preferred by the stakeholders was the scenario of self-regulated safety, while the current situation and safety culture scenarios were in general positively regarded as well (see Table 2 for ranking).

The scenarios least preferred by the stakeholders were Safe-by-Design and Design thinking. As mentioned above, the main reasoning for choosing self-regulated safety as the preferred scenario was based on the stakeholders’ view that a company’s self-interest already causes them to act cautiously with respect to the safety of its products. Moreover, there were concerns that additional rules from outside of the company would negatively influence the company’s flexibility. In general, the idea that companies would be able to implement their own internal procedures, but based on clear guidance from the government, comprising of a combination of different voluntary and supportive measures appealed most to the stakeholders. Additionally, some hybrid scenarios were suggested, including combinations of the scenarios on self-regulated safety and safety culture, and combinations of those two scenarios with the current situation scenario and/or safe-by-design. These hybrid scenarios were based on the idea that several elements from different scenarios might be combined to develop one solid safety framework, which potentially does not cause additional financial burden and is available for all companies that are interested in implementing such approaches. Of the eight interviewed plant breeders, four of them marked self-regulated safety and/or the current situation as their favorite scenarios, while three of them proposed the abovementioned hybrid scenarios comprising a combination between the current situation and (some) other scenarios. One plant breeder had Safe-by-Design as its favorite scenario.

### Proposed Safety Framework

Stakeholders voiced their preferences for a scenario that is self-regulated by the plant breeding sector as the preferred safety strategy to minimize hazards associated with the utilization of emerging technologies such as biotechnological innovations during crop breeding. The strategy should be relevant during all stages of development, voluntary, provide supportive measures, and preferably lead to little extra burden. Furthermore, it is preferable that such a strategy also synergizes well with existing internal protocols. However, some companies voiced their interest in guidance from the government, as part of a self-regulated scenario. A number of stakeholders suggested the possibility of integrating safety culture (*e.g*., based on ISO standards) and Safe-by-Design principles in a voluntary safety strategy. In such a scenario that is self-regulated, where the initiative is with the plant breeding sector, best practices for safe innovation are curated and developed by the sector in collaboration with independent researchers. Such an approach would be non-statutory and be separate from existing legal regimes. Complementary guidance and policy can be utilized to develop and update such a safety approach, when this is deemed necessary.

Based on the stakeholders’ input, namely a preference for self-regulation potentially in combination with safety culture aspects, we propose a non-statutory concept framework for safe innovation in crop breeding. We envision a framework that is voluntary and leads to minimal extra burden but can offer extra guidance where needed. This framework consists of different measurable safety levels that can be accredited using a certification system. Requirements for each safety level are freely accessible in the best practices that are agreed upon by the sector, and agreement as well as compliance can be assessed by independent organizations such as the NEN.

The safety culture ladder (SCL) initiative of the NEN, which encourages safety awareness and behavior to reduce unsafe situations, is an example of a safety strategy that is voluntary and that will result in minimal extra burden.^41^ The SCL was developed in 2012 by the Dutch organization ProRail and has since been implemented by around 2000 companies. Major changes were made to its updated version 2.0 in 2023 so that it became more broadly applicable, with references to railroads removed, for example. Its focus also shifted toward attitude, behavior, and corporate culture. Moreover, the concept has also become adopted in sectors beyond railroad and construction works, and by authorities and accrediting institutions worldwide, such as national/state worker health authorities (*e.g*., Australia) and international accrediting organizations such as Lloyd’s Register or Det Norske Veritas.^42,43^ Similar concepts for safety culture assessment tools have also been developed and implemented by others in parallel, such as the UK Health and Safety Executive’s “safety culture tool,”^44^ the energy sector’s Hearts and Minds program (originally started by Shell),^45^ and the application of the so-called “Hudson Framework” or “Hudson Ladder” to data management and occupational health.^46–48^ The SCL consists of five steps that describe safety awareness, attitudes and behavior. A higher step on this ladder means that safety is taken more seriously, with progressively increasing levels of trust, accountability, and information & transparency. The five different SCL steps are:

1. “pathological,” where there is little to no investment in improving behavior regarding safety,
2. “reactive,” where behavioral changes are ad-hoc and temporary,
3. “calculating,” where safety rules are deemed important,
4. “proactive,” where safety has great priority and is continually improved, and
5. “progressive,” where safety is fully integrated in the companies’ operational processes.

The NEN offers four different certified and non-certified products to assess safety behavior. Non-certified products are available for organizations who wish to start implementing the SCL on their own initiative, which consist of a self-assessment and SCL action plan. Certified products furthermore consist of annual audits performed by a certification body, which can then issue an obtained SCL certificate.

**Figure 2.**
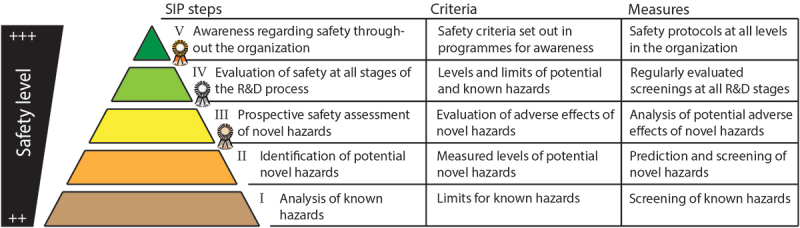
The proposed safe innovation in plant breeding framework, depicted step-wise in a pyramid. Each step represents one of five safety levels (I-V) and accompanying safety measures per level are shown. Certification schemes are available for levels III and higher, as indicated with a ribbon.

Our proposed safe innovation framework shares similarities with SCL in that we propose different levels of best practices and measures for stakeholders to follow regarding safety, with a focus on food safety. The proposed framework, which we coin as the “Safe Innovation in Plant breeding” (SIP, in Dutch “Veilig Innoveren in de Plantenveredeling [VIP]), consists of 5 levels, as depicted in the SIP pyramid (Figure 2). The levels vary from compliance to current regulations to a situation in which the stakeholder has detailed internal protocols in place and in which safety is an integral part of operational processes. This framework could be organized and kept up-to-date by relevant interest groups in collaboration with independent research institutes.

The proposed SIP framework consists of five safety levels (I-V, see Figure 2) starting with level I, the base of the pyramid. This level represents the current situation in which internal protocols compliant with relevant regulations are followed. Each higher level adds additional safety checks (as described in Table 3) to aid in the development of a safe end-product (*e.g*., food). Compared to other SCLs, the proposed SIP is conservative, with a certain degree of safety awareness already at level I. For example, reflecting the fact that safety/quality and customer-orientation is already engrained within the sector’s culture to a great extent.

**Table 3.** Description of SIP steps, related criteria and (food) safety measures.

SIP step	Criteria and additional safety measures
I.	Analysis and screening of known hazards (*e.g*., glycoalkaloids in novel potatoes) according to current regulation, standards, or limits for known hazards, as defined by appropriate guidance and regulation
II.	Identification of potential novel hazards (*e.g*., toxic components). Levels of such novel hazards are defined by prediction and screening methods. The practices described here may already be commonplace and part of internal/proprietary protocols. Adherence to requirements for step II can be demonstrated by means of self-assessment procedures and supporting guidance.
III.	Prospective safety evaluation of novel hazards and analysis of potential adverse effects. This can, for example, encompass theoretical assessments described in the most recent scientific literature, and development of experimental procedures to test identified hazards. Adherence to requirements for step III is evaluated by means of recurring audits and rewarded with a certificate.
IV.	This step consists of evaluations of safety at all stages of the R&D process and regular improvements of protocols when possible. Levels and limits of newly identified potential novel and known hazards are defined by means of monitoring and screening. Adherence to the requirements for step IV is evaluated by means of audits, and rewarded with a certificate.
V. *As safe as possible*	Step V encompasses the awareness of safety throughout the organization. All employees periodically participate in training and education programs regarding safety. Such safety programs, describing, relevant safety criteria are available for all levels in the organization. Adherence to requirements for step V is evaluated by means of audits, and rewarded with a certificate.

For each safety level within this framework, general information, including best practices containing relevant practical information, will be publicly available. Free access to this information will allow companies to experience less of a hurdle to implement measures when they deem this necessary. This may also benefit SMEs, as additional costs to get access to such information may pose relatively greater disadvantages for smaller businesses. Additionally, certification schemes relevant for each safety level are available, which may consist of audits (*e.g*., performed by a certification body such as NEN) and/or self-assessments similar to SCL.

Several of the mentioned criteria and measures described in the different SIP steps are, according to interviewed stakeholders, already common practice and part of internal protocols. Furthermore, steps IV and V in this proposed framework contain elements of Safe-by-Design principles and/or safety culture, in which safety is an integral part throughout the research and development (R&D) process and awareness is nurtured throughout the organization. This way safety is always considered and adhered to at all levels, from the work floor to the management layer of the respective organization.

This proposed SIP framework resonates with the preferences voiced by the stakeholders; it is mainly voluntary, and relevant guidance and best practices are curated by the sector in collaboration with independent researchers. It is imperative that guidance to reach each safety level is freely available for all stakeholders in the plant breeding sector to limit the administrative and financial burden. Additionally, SIP offers the opportunity to reach higher safety levels, which are rewarded with certifications. An official certification body such as the NEN could manage the certification of SIP. For transparency a database can be maintained listing all organizations that have achieved safety level III-V certifications.

## Discussion

Because of the increasing pace of biotechnological innovations in plant breeding, the aim of this study was to investigate, through stakeholder interviews, the feasibility of implementation of a pro-active safety framework for emerging biotechnological innovations for the Dutch plant breeding sector. For this purpose, we interviewed stakeholders of the Dutch plant breeding sector as well as governmental organizations and interest groups. It may be argued that plant breeding has an intrinsically low risk profile and there is therefore no need for a safety framework.^49^ Nevertheless, it is known that conventional plant breeding technologies can influence the formation of toxic compounds in traditional crops, such as psoralens in celery, and glycoalkaloids in Solanaceous crops (*e.g*., potato and tomato).^50^ Safe innovation systems may be of added value to evaluate at an early stage of development if the formation of such toxins has been affected by the application of emerging technologies. Moreover, future biotechnological innovations might be considerably diverse and carry yet unforeseen risks. Such unforeseen risks as well as biotechnological innovations in general may pose challenges for regulation and stewardship. An example of a challenge for stewardship comes from genome editing, where regulation mandates the traceability of small genomic alterations, which may not be possible with current detection methods.^13^ This poses additional hurdles for product development and commercialization. Unforeseen effects may affect safety and, therefore, it can be of added value to have a system in place with guidance for safety evaluation.^51^ Such a system can be used to keep track of developments in biotechnology and stay up to date with best practices focused on safety (*e.g*., environmental, or food safety), ahead of the introduction of any legislation regulating the safety evaluation and market approval of crops emerging from these developments.

Among stakeholders, a clear preference for a scenario in which safe innovation is self-regulated became evident. It was mentioned that a self-regulation scenario may lead to the least additional burden for the sector and can sufficiently aid the safe development of novel plant varieties. Likewise, stakeholders argued that the scenario of maintaining the status quo will not lead to an additional burden for the sector. Stakeholders also agreed that the opportunity to consult with experts may be valuable, in particular for SMEs which may not have access to this expertise yet within their own organization. Such a consultation process may offer the possibility to get into contact with experts such as toxicologists, but also with advisors specialized in ethics and sustainability.

Based on this input, we envision the voluntary SIP framework. The proposed SIP framework (Figure 2) contains five different measurable safety levels. Compliance with the framework at any level may also be accredited using a certification system. Requirements for each safety level are freely accessible in the best practices that are agreed upon by the sector, and agreement as well as compliance can be assessed by independent organizations. This promotes the availability of guidance for all interested businesses (SMEs as well as larger corporations). Additionally, interested stakeholders may opt to follow higher certification levels on a voluntary basis. However, additional certification and auditing of companies requires extra investments. Therefore, certification schemes and audits may be challenging for SMEs, as this can lead to additional bureaucracy and financial hurdles. Current practice of implementing the “safety ladder” in other sectors shows that various types of assessments are possible, including the following:^52^

- A self-assessment in the first year, followed by checks of the action plan in the 2^nd^ and 3^rd^ years; a document will be issued stating that the necessary steps have been followed.
- A “light” type of assessment with a partial audit in the 1st year and follow-up action plan checks in the 2^nd^ and 3^rd^ years; a statement will be issued with an indication of the step reached on the ladder.
- Assessments with a complete annual audit in the first year, and partial (40%) or complete (“original” mode) ones in the 2^nd^ and 3^rd^ years; a certificate will be issued in both cases.

Empirical and anecdotal reports from other sectors indicate that ladder-based approaches foster improvement of safety awareness within organizations. For example, these sectors include the gas & petroleum and trucking sectors.^53,54^

Moreover, it is anticipated that our proposed SIP framework also harmonizes well with ELSA and RRI-based approaches due to its intended focus on safety awareness within the organization as well as opportunities for continuous improvement of internal procedures. Also, SIP synergizes well with the four core features of RRI, namely:^31^

1. *anticipation*, the SIP framework is focused on identifying and minimizing potential risks of emerging technologies already during early development stages for the benefit of crop breeding innovation.
2. *inclusiveness*, SIP is intended to be non-statutory and self-regulated by the plant breeding sector, whereby collaboration with independent organizations is possibles. This empowers stakeholders to shape and improve the framework according to needs from, for example, the sector or from society.
3. *reflexivity*, because the plant breeding sector is responsible for the SIP framework, this stimulates stakeholders from the sector to take responsibility for the potential impact of their innovations in society.
4. *responsiveness*, the SIP framework intended to be non-statutory and is therefore not beholden to the bureaucracy of drawing official regulations. This allows SIP to be adapted in a more flexible way to new circumstances, insights, and surprises

Additional practices focusing on participatory stakeholder engagement can be part of a broader SIP approach containing ELSA and RRI concepts.^3,30^

On a similar note, Bonse and coworkers reported the House of Total Safety Culture (HSC) standard of guidelines for the agri-food sector.^55^ The HSC standard is developed for the entire value chain of the agri-food sector and is based on SCL as well as ISO 9001. As with SIP, the HSC standard focuses on safety culture and continuous improvement of processes. Similarly, it intends to implement assessment and levels of certification, which are core elements of SCL. Bonse et al. argue that for such a standard to be effective, entry barriers for organizations should be kept as low as possible. Furthermore, HSC guidelines should support organizations and motivate them to reach the next level. Communication of milestones and successes can likewise incentivize aiming for higher HSC levels. The first HSC pilots projects have been started within the agri-food industry.^55^ Compared to HSC, the SIP framework focuses specifically on the R&D part of plant breeding, by providing best practices and safety strategies.

A voluntary framework such as SIP can also be of added value to the plant breeding sector, in particular regarding early preparedness for market approval, and potentially increase consumer acceptance. Furthermore, a SIP framework may help future procedures for regulatory approval when products developed using novel crop breeding innovations move to the world market. Adherence to such safe innovation best practices can aid in the compilation of a safety dossier, as part of current or future requirements for market approval, when necessary. Best practices described in SIP can for example also be of added value for future market approval applications for NGT crops under the proposed NGT regulation in the EU. This also holds true for the fulfillment of policy goals to achieve product safety also when there is no clarity yet on the regulation of a novel technology, such as under the Dutch national environmental policy framework. The government calls upon companies to assume their responsibility and to choose quality over quantity, whilst intending to collaborate with them to develop new business models that focus more on the provision of services instead of products.^56^ Such Safe-by-Design principles can also benefit a crop breeding innovation approach that follows our proposed SIP framework. Indeed, certain Safe-by-Design elements are already partly incorporated in the envisioned SIP steps IV and V.

## Concluding Remarks

Crop breeding innovations are advancing rapidly and can be used to produce enhanced plant varieties with specific desired traits. Often, these new crops may not be fundamentally different from those created using conventional methods, especially when the techniques result in changes indistinguishable from natural genetic variations. However, the use of such crop breeding innovations may pose unforeseen risks, including unintended alterations alongside the intended ones.

The development of additional internal safety protocols in the plant breeding sector, such as the proposed voluntary “Safe Innovation in Plant breeding (SIP)” framework in this paper, may complement existing plant breeding practices. Thus, safety protocols can help to maintain an excellent standard of food safety when new techniques are applied that are not covered by current and proposed GMO regulations.

During interviews and workshops, current safety strategies in plant breeding as well as potential safety scenarios were discussed with stakeholders, with a focus on food safety. Some key findings of this study included:

1. Biotechnological innovations are of added value for the plant breeding sector, for use in research and development but also for the development of novel plant varieties. Such tools could potentially allow for precise and quick genetic alterations, compared to conventional tools, greatly speeding up the overall process.
2. Stakeholders expressed a preference for a “self-regulated” safety scenario over scenarios of Safe-by-Design, safety culture, design thinking, and the current Dutch situation. In the self-regulated safety scenario, the initiative in developing best practices lies with the plant breeding sector, in collaboration with independent researchers. Stakeholders argued that such a scenario would be most beneficial for companies, as it allows them to adopt their own internal procedures. Such procedures could be based on clear guidance from the government, comprising of a combination of different voluntary and supportive measures.
3. The tentative SIP framework is proposed by us as a non-statutory means to assist the plant breeding sector with safe innovation and regulatory preparedness. SIP is a voluntary framework consisting of five different “safety levels.” It shares similarities with the safety culture ladder approach of the NEN (NEN, 2024b). In this framework, the initiative is with the plant breeding sector and its implementation could be organized by relevant interest groups in collaboration with independent research institutes. Our opinion is that this SIP framework, the accompanying general guidance, and best practices should be publicly available to interested stakeholders.

Taken together, safety strategies such as the proposed SIP framework can be of added value to the plant breeding sector and regulators alike. A well-structured framework can help the sector by promoting innovation and anticipating changes to the regulatory situation. Likewise, such a voluntary framework can help regulators as safety requirements set out in the framework will contribute to safe food and therefore aid in safeguarding public health for biotechnological inventions that are not yet or incompletely covered by specific legislation.

## Supplementary Material

Supplementary Information.docx
