# Supplementary material for: Feasibility of a safe innovation framework for crop breeding
Source: GM Crops Food. 2025 Jun 26;16(1):498–515. doi: 10.1080/21645698.2025.2524236 (PMC12203842; doi:10.1080/21645698.2025.2524236)
Supplement: Supplementary Information.docx [file KGMC_A_2524236_SM9012.docx]

Supplementary information

Stakeholder engagement activities for this study consisted of interviews and workshops (for more information see: <https://research.wur.nl/en/activities/safe-innovation-in-crop-breeding-2>).

Interviews questions and case studies (**A**), as well as the workshop programme (**B**) are provided below.

**A1 Interview questions:**

*Technological developments*

- Which historical technological developments, since ca. the 90s, have according to you been the most important for plant breeding? And do you have any specific examples?
- Which more recent (biotechnological) innovations are, according to you, important for the plant breeding sector and may potentially be used on a large, in the short term?
- Which (bio)technological innovations are already used by you in the breeding process? Do perform these technologies in house or are these outsourced?
- Do you foresee potential problems for safety of a new plant variety when (bio)technological innovations are utilized? If yes, what kind of issues?

***Current safety strategies, internal protocols***

- How does (food) safety have a place within your company now, and how is attention paid to this in the development of new plant varieties?
- What do you test for, within your company, to guarantee the safety of a new plant variety?


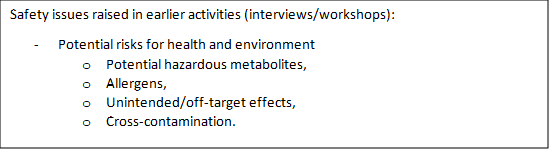


- How do you test to see if a new variety is as safe as possible?

- Which safety strategies and internal protocols are being followed within your company already in the development of new plant varieties?
- Have you previously noticed unforeseen hazards during the development of new plant varieties, despite internal safety measures? And how did you deal with this then?

***Safe-by-Design, safety culture, other concepts***

- What do you think should be included in a safety strategy to support the development of new plant varieties?
- Do you think that a safety strategy, for example based on Safe-by-Design, should be regulated by the government?
- In addition to safety, the possibility of consultation with risk professionals and experts from the relevant authorities can also be part of a broad Safe-by-Design concept. Are you interested in such a possibility of consultation? And what would you like to discuss in such a consultation?

**Potential scenarios for safe innovation**

The following table provides a brief description of various scenarios for ensuring safety when using biotechnological innovations. Such scenarios can be important for the sector to respond in a timely manner and to be prepared for any future regulatory changes. A detailed description of the different scenarios can be found at the bottom of this document.

Could you indicate which of these scenarios you prefer (yellow column “Ranking”)? Please also indicate which parts of the other scenarios you also assess positively. For example, you can also combine parts into a “hybrid” scenario/make your own suggestions that meet your preferences (you can enter this in the last yellow row “possible hybrid scenario/own suggestion”).

| **Scenario** | **Regulated by** | **Applicability “scope”** | **Extra burden?** | **In line with or compatible with current strategy?** | **Ranking (1-5)** |
| --- | --- | --- | --- | --- | --- |
| **1 Safe-by-Design** | Legal | From concept to commercialization | Possible, if security strategy deviates significantly from current internal protocols | Adaptable, depending on compliance with currently used safety protocols |  |
| **2 Self regulated safety** | Business driven | During research and development process | Somewhat, perhaps agreement with current internal protocols | Adaptable, and perhaps in line with current strategy |  |
| **3 Safety culture** | Business driven /legal | Safety awareness in the organization, including research/development (for example according to ISO standards), broader than just safety during the development of new products | To some extent, is often already part of company-wide “safety thinking” according to ISO standards | In accordance with current standards such as ISO |  |
| **4 Design Thinking** | Business driven /legal | From concept to commercialization, incl. Prototyping according to Design thinking stages | Possibly, may require different internal attitude and mindset | Adaptable, depending on agreement with the current situation and possibly a different mindset |  |
| **5 Huidige situatie** | Business driven | Security strategy according to internal protocols | No, continuation of current situation | Not applicable |  |
| **possible “hybrid” scenario/own suggestion** |  |  |  |  |  |

Additional explanation/remarks: ……

**Case studies**

We would be happy to discuss case studies with you of technologies that could be applied in plant breeding. How would you handle the evaluation of the safety of the resulting crops/products within your organization?

**Case study 1: *de novo* domestication**

Wild crops, or for example ancestors of current domesticated crops, can be domesticated via *de novo* domestication. Biotechnological innovations can contribute to this process of *de novo* domestication.

By means of such a *de novo* domestication strategy a related wild variety of for example a tomato^1^ with “wild” characteristics can be domesticated.

- How would you evaluate such a domesticated plant according to your internal way of working? (for example, if you have developed such a plant in your company)
- In what way would you take into account possible unintended effects of the technology used for *de novo* domestication?
- Which analyses would be required for this?
- Could you call this "Safe-by-Design"? If yes, please explain your answer. If not, what would need to be changed about your working method to be able to call it that?

**Case study 2: Transgenic rootstock grafting**

Robust plant varieties can be obtained by placing a non-GMO graft on genetically modified rootstocks. Recent research by, among others, the company EpiCrop shows that GMO rootstocks in which genes that play a role in making epigenetic changes have been modified also influence the epigenome of the graft. These epigenetic changes are inherited to at least five generations^2^.

This rootstock grafting technology could be used to create new varieties with, for example, epigenetic adjustments.

- How would you evaluate such a rootstock grafted plant according to your internal way of working? And how would you judge products (fruits, seeds, or other edible parts) from such varieties? (for example, if you have developed such a plant in your company)
- Would you conduct such an analysis yourself or outsource it to another organization?
- In what way would you take into account possible unintended effects of the technology used for de novo domestication?
- Which analyses would be required for this?
- Could you call this "Safe-by-Design"? If yes, please explain your answer. If not, what would need to be changed about your working method to be able to call it that?

**B Workshop program safe innovation in crop breeding, as shared with stakeholders**

*Invitation to an online workshop on safety aspects and Safe-by-Design principles for innovations in crop breeding*

Biotechnological innovations are being developed rapidly and can subsequently find application in crop breeding. Techniques such as genome editing as well as epigenome editing are examples of such technological innovations. However, adaptations of European regulations to the situation created by these new technologies are introduced at a slow pace. The development of a safety framework based on the Safe-by-Design principle can aid the safety guarantee of biotechnological innovations in anticipation of such changes to the current regulatory situation. In this workshop we will discuss safety aspects associated with biotechnological innovations, current safety practices, as well as the potential application of Safe-by-Design best practices. This workshop is part of a policy-supporting project on safety aspects regarding the application of biotechnological innovations in crop breeding.

This project aims to list safety aspects in crop breeding, such as:

1. currently employed safety strategies,
2. potential improvements to the current situation regarding safety, and
3. the need for a general Safe-by-Design strategy.

In this workshop we will:

- share information about Safe-by-Design,
- list current safety practices,
- list needs and bottlenecks regarding safety aspects and Safe-by-Design,
- discuss safety strategies for biotechnological innovations.

*Program:*

11:00 Welcome & Introduction

11:15 Presentation about Safe-by-Design,

12:00 Plenary discussion about safety strategies

12:45 Break

13:00 Discussion in break-out groups

13:45 Plenary discussion outcomes & closure

*For whom is this workshop intended?*

Stakeholders in the plant breeding sector who work with or (indirectly) deal with crop breeding and the application of biotechnological innovations. Feel free to share this invitation with interested colleagues.
